# Efficacy of functional alignment in total knee arthroplasty in restoring in vivo cruciate ligament forces and knee kinematics compared with mechanical alignment

**DOI:** 10.1002/ksa.70151

**Published:** 2025-10-27

**Authors:** Kenichi Kono, Hiroshi Inui, Tomofumi Kage, Takaharu Yamazaki, Shuji Taketomi, Darryl D'Lima, Tetsuya Tomita, Sakae Tanaka

**Affiliations:** ^1^ Department of Orthopaedic Surgery, Faculty of Medicine The University of Tokyo Tokyo Japan; ^2^ Department of Orthopaedic Surgery Saitama Medical University, Saitama Medical Center Saitama Japan; ^3^ Department of Orthopaedics Tokyo Teishin Hospital Tokyo Japan; ^4^ Department of Informatics Faculty of Informatics, Shonan Institute of Technology Kanagawa Japan; ^5^ Department of Molecular Medicine Arthritis Research The Scripps Research Institute San Diego California USA; ^6^ Department of Orthopaedic Biomaterial Science Osaka University Graduate School of Medicine Osaka Japan; ^7^ Department of Health Sciences, Graduate School of Health Sciences Morinomiya University of Medical Sciences Osaka Japan

**Keywords:** cruciate ligament force, functional alignment, high‐flexion activities, normal knee, total knee arthroplasty

## Abstract

**Purpose:**

The impact of coronal alignment on cruciate ligament forces in bicruciate‐retaining total knee arthroplasty (TKA) remains unclear. We aimed to clarify in vivo cruciate ligament forces and knee kinematics among mechanically aligned (MA) and functionally aligned (FA) TKAs and normal knees.

**Methods:**

Normal, MA TKA and FA TKA knees were assessed during squatting using fluoroscopy. Tibiofemoral kinematics was measured using a two‐dimensional/three‐dimensional registration technique, including axial rotation, varus‐valgus angle, and anteroposterior translation (APT) of the surgical epicondylar axis (SEA) and low contact points (LCPs). Ligament tensions during knee flexion were analysed in the anteromedial anterior cruciate ligament (aACL) and posterolateral anterior cruciate ligament (pACL) and anterolateral posterior cruciate ligament (aPCL) and posteromedial posterior cruciate ligament (pPCL).

**Results:**

Both MA and FA TKA knees exhibited greater external femoral rotation and less varus alignment. Medially, MA TKA knees were positioned more anteriorly at 0° flexion. Laterally, both TKA knees were positioned more posteriorly up to 10° flexion. Medially, MA TKA knees were positioned more anteriorly at 0° flexion. Laterally, both TKA knees were positioned more posteriorly up to 10° flexion. In lateral LCPs, both TKA knees were more anteriorly positioned. aACL and pACL tensions decreased with flexion, with no significant differences between TKA and normal knees, whereas aPCL and pPCL tensions increased with flexion. Beyond 80° flexion, MA TKA knees exhibited greater aPCL tension. Beyond 90° flexion, pPCL tension was higher in MA TKA knees.

**Conclusions:**

PCL tension was notably higher in MA TKA knees. However, FA TKA and MA TKA demonstrated comparable kinematics and changes in ACL force. Although FA TKA more closely restored the physiological balance of the cruciate ligaments, the overall effect appeared to be limited.

**Level of Evidence:**

Level II.

**Abbreviations**
2D: two dimensional
3D: three dimensional
aACL: anteromedial anterior cruciate ligament
ACL: anterior cruciate ligament
aPCL: anterolateral posterior cruciate ligament
APT: anteroposterior translation
BCR: bicruciate‐retaining
CAD: computer‐aided design
CR: cruciate ligament retaining
CT: computed tomography
FA TKA: functional alignment total knee arthroplasty
LCP: low contact point
MA TKA: mechanical alignment total knee arthroplasty
MRI: magnetic resonance imaging
OA: osteoarthritis
pACL: posterolateral anterior cruciate ligament
pPCL: posteromedial posterior cruciate ligament
PROM: patient‐reported outcome measure
SEA: surgical epicondylar axis

## INTRODUCTION

Sacrificing an intact anterior cruciate ligament (ACL) during posterior‐cruciate ligament retaining (CR) total knee arthroplasty (TKA) has been associated with reduced patient satisfaction [14]. Additionally, ACL‐sacrificing TKA often results in paradoxical anterior tibial movement [4], indicating that such designs fail to restore normal knee kinematics [1]. By contrast, bicruciate‐retaining TKA (BCR‐TKA) may better replicate physiological kinematics and improve patient‐reported outcome measures (PROMs) [18]. Previous studies have suggested that mechanical alignment (MA) BCR‐TKA with an anatomical articular surface can facilitate more natural knee motion [18, 19, 20, 21]. However, native knee kinematics are not fully restored following MA BCR‐TKA [44]. Further studies have demonstrated a significant postoperative increase in ACL and posterior cruciate ligament (PCL) forces in MA BCR‐TKA knees [16, 17]. Furthermore, an in vitro study demonstrated that the ACL in MA BCR‐TKA knees undergoes higher tension and altered kinematics during passive flexion‐extension, differing from its function in intact knees [29].

MA remains the gold standard for TKA, with well‐documented long‐term clinical outcomes and implant longevity [10]. However, approximately 10%–20% of patients report dissatisfaction with their surgical outcomes [3, 28, 41]. To address this issue, alternative alignment methods have been introduced [30]. One such approach, functional alignment (FA), considers ligament balance intraoperatively through navigation or robotic assistance [30]. In BCR‐TKA, FA has demonstrated excellent postoperative clinical outcomes [11]. Kinematic analysis has shown that FA TKA exhibits medially stabilised kinematics [15]. However, whether various coronal alignment strategies can restore normal knee kinematics and cruciate ligament forces remains unclear.

High knee flexion activities, such as gardening and exercising, are common in daily life. Several studies have associated participation in these activities with improved clinical outcomes, greater patient satisfaction, and better alignment with patient expectations following knee joint replacement [27, 28]. Consequently, this study focused on evaluating high knee flexion activities.

This study aimed to compare in vivo cruciate ligament forces and kinematics between MA and FA TKA knees and those in normal knees during high‐flexion activities. We hypothesised that FA TKA would more closely replicate the kinematics and cruciate ligament forces observed in normal knees.

## METHODS

This prospective cohort study analysed 57 knees from 44 individuals, including 20 knees from 10 healthy volunteers, 19 knees from 17 patients who underwent MA TKA (Journey II XR; Smith & Nephew), and 18 knees from 17 patients who underwent FA TKA. All BCR‐TKAs were performed to treat bicompartmental or tricompartmental osteoarthritis (OA) (Kellgren and Lawrence grade III or Ⅳ) with an intact ACL. ACL integrity was confirmed preoperatively using magnetic resonance imaging (MRI). All participants provided informed consent, and the study was approved by the Institutional Ethics Review Board of the University of Tokyo (approval number: 10462‐(2)). Patients aged between 20 and 60 years, with absence of knee symptoms, and with no radiographic evidence of OA on computed tomography (CT) comprised the normal knee cohort. Patients scheduled for BCR‐TKA were recruited if they were able to perform squatting activities preoperatively. The inclusion criteria for the BCR‐TKA group were bicompartmental or tricompartmental OA with an intact ACL. Patients with severe valgus deformity (hip‐knee‐angle ≥190°) and a history of joint trauma were excluded.

The surgical procedures were performed by five knee surgeons, with a highly experienced surgeon (HI) overseeing all procedures as either the chief surgeon or first assistant. In MA TKA, coronal bone resections of the distal femur and proximal tibia were aligned perpendicular to the mechanical axis. In FA TKA, the distal femoral cut was made to replicate prearthritic condyle thickness. Varus‐valgus stress was applied intraoperatively to evaluate medial and lateral joint laxity under navigation, and the proximal tibial cut was adjusted accordingly [11, 15]. The tibial design of the JOURNEY TKA system was also asymmetric. The thinnest tibial insert measured 8.5 mm on the medial side and 11 mm on the lateral side. Accordingly, in varus knees, the amount of bone resection at the lateral tibial plateau was standardized at 11 mm, whereas the medial resection varied from 5 to 9 mm depending on soft tissue balance at that stage [12]. In the sagittal plane, the navigation system was used to reproduce the native slope in patients with a posterior tibial slope of <10°. In patients with a posterior tibia slope of >10°, the slope was reduced to ≤10° to avoid excessive ACL stress [12].

Study participants performed a squatting activity while fluoroscopic surveillance was conducted in the sagittal plane. Each participant performed the motion at a self‐selected pace, practicing several times before the recording. Knee motion was assessed at least 6 months postoperatively following BCR‐TKA. The mean follow‐up duration was 10.8 ± 4.5 months. Participant characteristics and PROMs, including the Knee Injury and Osteoarthritis Outcome Score [32] and the 2011 Knee Society Score [34], are presented in Tables 1 and 2. The mean hip–knee–ankle angles at the time of analysis were 177.9 ± 2.0° in MA TKA and 177.8 ± 2.7° in FA TKA. Radiographic component positioning was evaluated using the Knee Society TKA Roentgenography Evaluation [5]. In the anteroposterior view, the mean femoral component angles were 99.8 ± 2.4° (MA TKA) and 98.5 ± 1.7° (FA TKA) (*α* angle), whereas the mean tibial component angles were 86.8 ± 1.8° and 87.6 ± 1.5° (*β* angle), respectively. In the lateral view, the mean femoral component alignment angles were 2.6 ± 1.7° (MA TKA) and 2.0 ± 1.4° (FA TKA) flexion (γ angle), whereas the tibial posterior slopes were 3.9 ± 2.1° and 5.6 ± 1.9°, respectively.

**Table 1:** Participant's characteristics.

	Normal	UKA	BCR‐TKA
Age (years)	34.5 ± 2.5	73.2 ± 6.5	72.3 ± 5.9
Body height (cm)	174.0 ± 0.1	154.6 ± 9.7	157.4 ± 6.9
Body weight (kg)	69.8 ± 7.7	57.9 ± 7.7	60.2 ± 7.9
Follow‐up duration (months)	NA	9.5 ± 2.3	8.1 ± 8.2
Men/Women	20/0	6/11	3/12

Abbreviation: BCR‐TKA, bicruciate‐retaining total knee arthroplasty.

**Table 2:** PROMs (postoperative 1 year).

	MA‐TKA	FA‐TKA	*p* value
Knee Injury and Osteoarthritis Outcome Scores (each 100 points)			
Pain	86.0 ± 12.0	88.9 ± 8.8	0.48
Symptoms	83.5 ± 9.9	88.9 ± 7.6	0.13
Function in daily living activities	86.8 ± 10.8	90.3 ± 9.5	0.28
Function in sports and recreation	61.1 ± 26.2	65.0 ± 21.8	0.77
Quality of life	72.4 ± 21.8	76.7 ± 16.7	0.57
Knee Society Score 2011			
Symptoms (25 points)	20.7 ± 3.2	21.1 ± 3.4	0.68
Satisfaction (40 points)	30.6 ± 7.6	31.3 ± 6.6	0.80
Expectation (15 points)	10.4 ± 2.3	10.6 ± 2.7	1.00
Functional activities (100 points)	77.5 ± 17.7	79.1 ± 15.7	0.91

Abbreviations: FA‐TKA, functional alignment total knee arthroplasty; MA‐TKA, mechanical alignment total knee arthroplasty; PROM, patient‐reported outcome measure.

**Figure 1:**
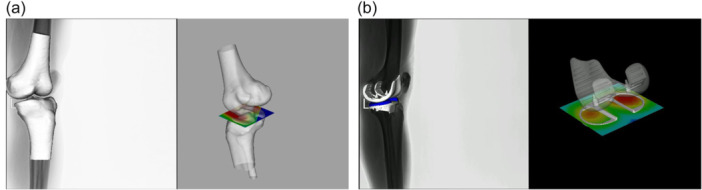
APT of femoral LCPs on the proximal tibial plane. (a) Normal knee. (b) TKA knee. APT, anteroposterior translation; LCPs, low contact points; TKA, total knee arthroplasty.

Knee motions were recorded as digital X‐ray images (1024 × 1024 × 12 bits/pixel, 7.5‐Hz serial spot images in Digital Imaging and Communications in Medicine format) using a 17‐inch flat panel detector system (ZEXIRA DREX‐ZX80; Toshiba). All images underwent dynamic range compression to enhance edge contrast. A two‐dimensional (2D)/three‐dimensional (3D) registration technique was employed to determine the spatial position and orientation of the knee [24, 42]. This technique utilises a contour‐based registration algorithm that integrates single‐view fluoroscopic images with 3D computer‐aided design (CAD) models [24, 42]. Preoperatively, CT scans were used to generate 3D bone models, which served as CAD models. In normal knees, 2D fluoroscopic images were integrated with a 3D model generated by a semi‐automated algorithm from the preoperative CT scan. In BCR‐TKA knees, extracting precise bony contours from CT scans proved challenging due to metal artifacts. To mitigate this, 2D/3D registration of the femoral and tibial implants was initially performed, followed by 2D/3D registration of the femoral and tibial bone models. The postoperative relationship between the implant and bone was then corrected using postoperative CT, as previously described [17, 18]. The relative position between the implant and bone was aligned using surface registration techniques that matched preoperative 3D models with postoperative 3D reconstructions derived from CT scans [17, 18]. The reported accuracy rates of relative motion estimation between 3D bone models were ≤1° for rotation and ≤1 mm for translation [24]. Local coordinate systems were established within the bone models, following previously described methodologies [24]. Knee rotations were computed using the joint rotational convention established by Grood and Suntay [7]. The femoral rotation and varus‐valgus angle relative to the tibia, as well as the anteroposterior translation (APT) of the surgical epicondylar axis (SEA)—defined as the line connecting the medial sulcus (medial side) and lateral epicondyle (lateral side) of the femur on a plane perpendicular to the tibial mechanical axis—were analysed at each flexion angle [20, 24]. Additionally, the APT of low contact points (LCPs) of the femur on the proximal tibial plane was assessed. The APT of the SEA was calculated as a percentage relative to the AP dimension of the proximal tibia [20, 24]. The APT of LCPs was assessed as the variation beyond 0° of flexion (Figure 1). External rotation was considered positive, whereas internal rotation was considered negative. Similarly, valgus was defined as positive and varus as negative. APT values were considered positive when located anterior to the tibial axis and negative when located posterior. All values were expressed as the means ± standard deviations.

**Figure 2:**
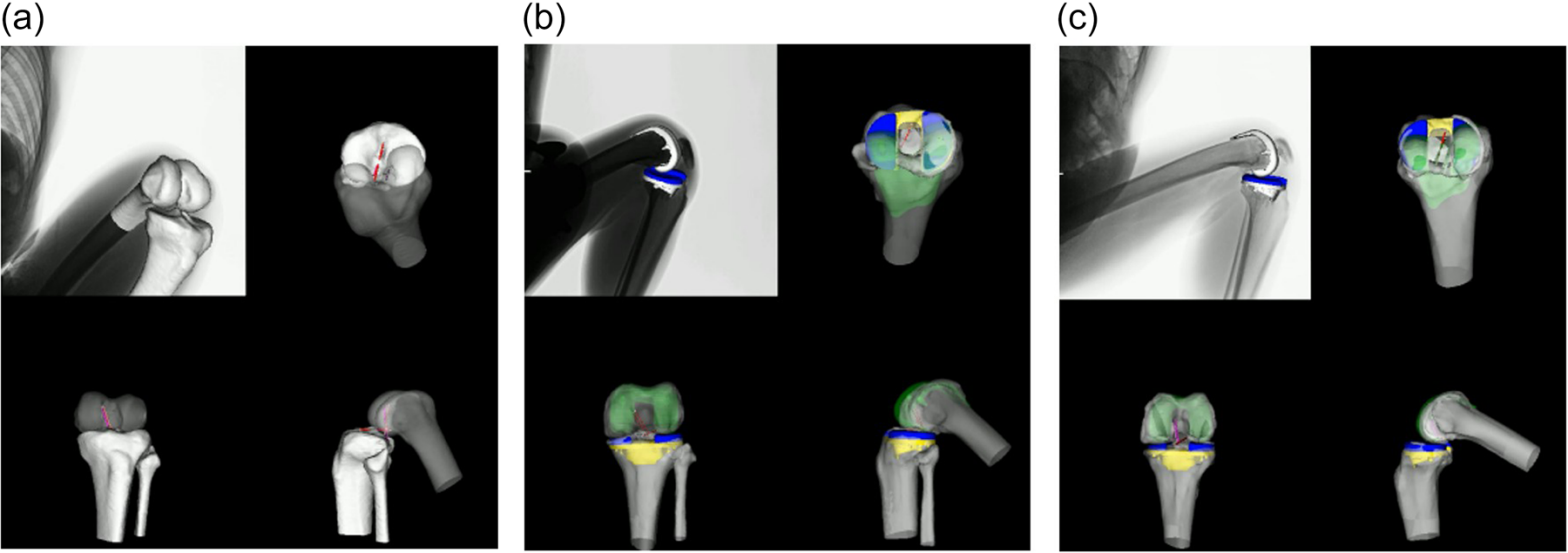
2D/3D registration and ligament force evaluation. (a) Normal knee. (b) MA TKA knee. (c) FA TKA knee. 2D/3D, two‐dimensional/three‐dimensional; FA TKA, functional alignment total knee arthroplasty; MA TKA, mechanical alignment total knee arthroplasty; TKA, total knee arthroplasty.

The anteromedial and posterolateral bundles of the ACL (aACL and pACL) and the anterolateral and posteromedial bundles of the PCL (aPCL and pPCL) were identified using osseous landmarks on preoperative CT and MRI scans [9, 23, 25, 38]. In BCR‐TKA knees, preoperative CT and MRI were utilised to confirm the ligament attachment sites [16, 17]. The accuracy of the ligament attachment area was within 0.7 ± 0.1 mm [25]. Ligament strains and forces for the cruciate ligament bundles were calculated using commercially available software (VivoSim; Advanced Mechanical Technology Inc.). Ligament paths were modelled as straight‐line segments, and the effects of ligament‐bone contact were not considered. Each ligament was assumed to exhibit elastic properties, with its mechanical behaviour characterised using a nonlinear force–strain relationship [2, 31, 35]. The stiffness values of the model ligaments were derived from previously published data [16, 35, 36] and further adjusted to match knee‐joint laxity measurements from intact and ACL‐deficient knees as reported in cadaveric studies [31, 37]. Cruciate ligament forces at each flexion angle were computed for each squatting cycle (Figure 2). Changes in kinematics and cruciate ligament forces in normal knees and BCR‐TKA knees were analysed with respect to flexion angle.

### Statistical analysis

Statistical analyses were conducted using IBM SPSS Statistics for Windows, version 26.0 (IBM Corp.) (2017). A two‐way analysis of variance was performed to assess the differences between normal knees and BCR‐TKA knees, followed by post hoc pairwise comparisons using the Dunnett test. A *p* ≤ 0.05 was considered significant. A priori power analysis was conducted using G*Power (version 3.1.9.7; Heinrich Heine University) [6] prior to the study. The analysis indicated that a minimum of 18 knees was required to achieve an alpha level of 0.05, a power of 0.8, and an effect size of 0.25.

## RESULTS

### Kinematic changes

During squatting, normal knees flexed from −6.7 ± 4.7° to 154.3 ± 4.1° on average. In the BCR‐TKA group, MA TKA knees flexed from −3.6 ± 4.5° to 118.1 ± 12.5°, whereas FA TKA knees flexed from −1.5 ± 4.1° to 118.7 ± 19.1°. The maximum knee extension angle in normal knees was significantly greater than that in FA TKA knees (*p* ≤ 0.01) but not significantly different from postoperative BCR‐TKA knees. The maximum knee flexion angle in normal knees was significantly higher than those observed in both MA TKA and FA TKA knees (*p* ≤ 0.01).

**Figure 3:**
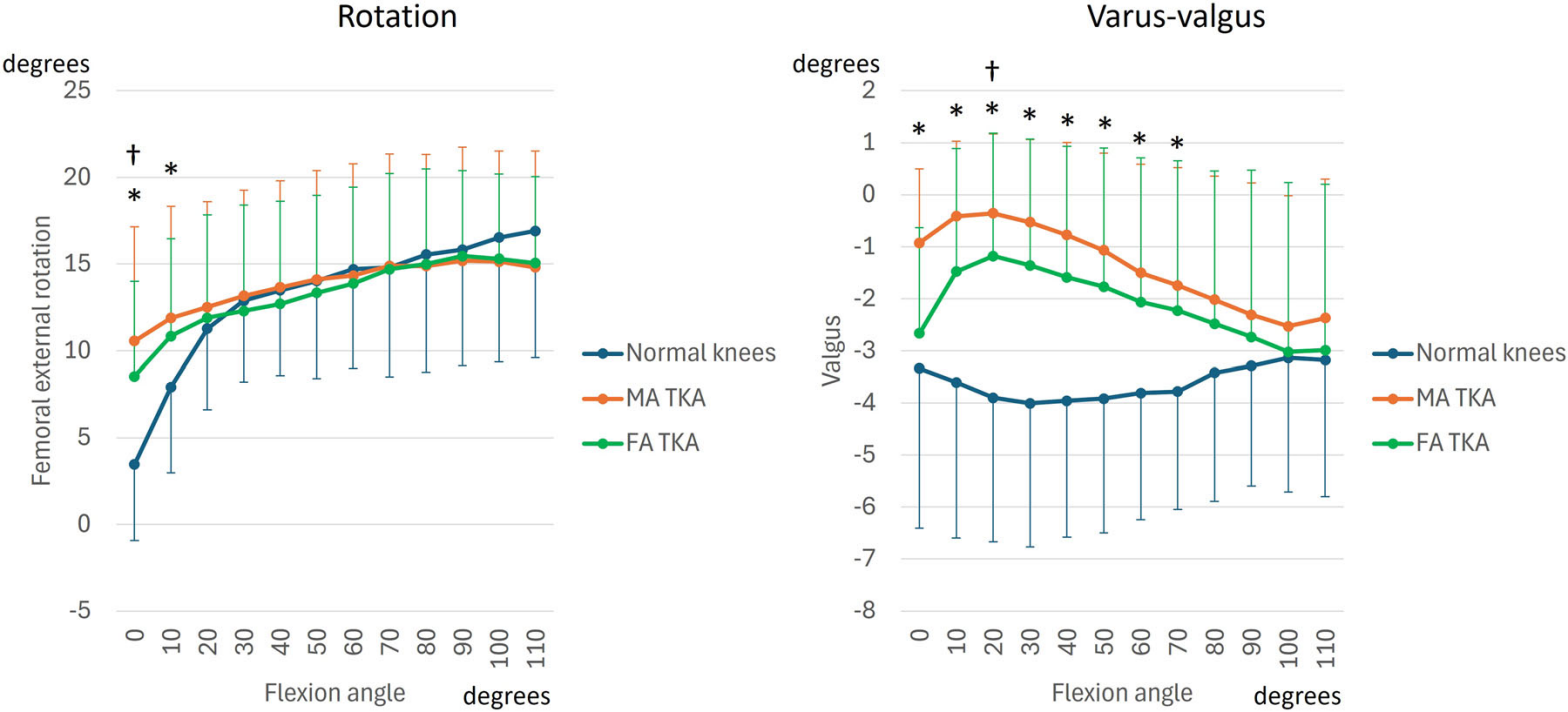
Rotation angle and varus‐valgus angle during squatting. The femur exhibited external rotation with flexion. *Significant differences between normal knees and MA TKA knees (*p* ≤ 0.05). †Significant differences between normal knees and FA TKA knees (*p* ≤ 0.05). FA TKA, functional alignment total knee arthroplasty; MA TKA, mechanical alignment total knee arthroplasty.

**Figure 4:**
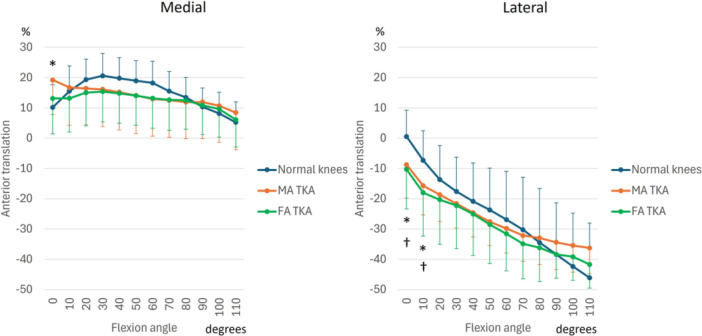
APT of the SEA during squatting. AP translation is expressed as a percentage relative to the AP tibia length. *Significant differences between normal knees and MA TKA knees (*p* ≤ 0.05). †Significant differences between normal knees and FA TKA knees (*p* ≤ 0.05). APT, anteroposterior translation; FA TKA, functional alignment total knee arthroplasty; MA TKA, mechanical alignment total knee arthroplasty; SEA, surgical epicondylar axis.

**Figure 5:**
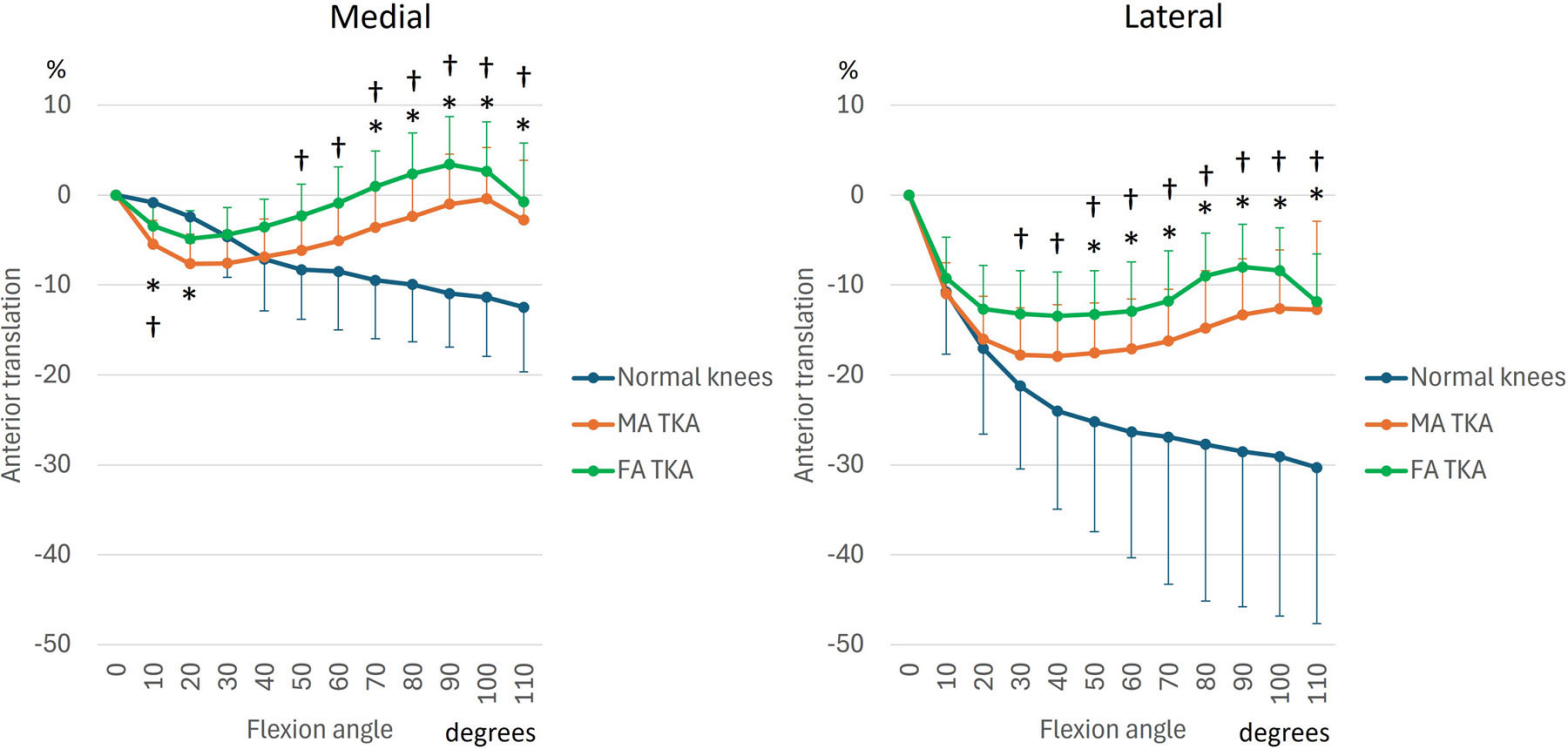
APT of the LCPs during squatting. *Significant differences between normal knees and MA TKA knees (*p* ≤ 0.05). †Significant differences between normal knees and FA TKA knees (*p* ≤ 0.05). APT, anteroposterior translation; FA TKA, functional alignment total knee arthroplasty; MA TKA, mechanical alignment total knee arthroplasty.

MA TKA knees exhibited greater external femoral rotation compared with normal knees at 0° and 10° of flexion. Similarly, FA TKA knees displayed greater external femoral rotation compared with normal knees at 0° flexion (Figure 3). Both MA TKA and FA TKA knees exhibited less varus alignment compared with normal knees (MA: up to 70° of flexion; FA: at 20° of flexion) (Figure 3). On the medial side of the SEA, MA TKA knees were positioned more anteriorly compared with normal knees at 0° of flexion (Figure 4). On the lateral side of the SEA, both MA and FA TKA knees were more posteriorly located compared with normal knees up to 10° of flexion (Figure 4). In medial LCPs, both TKA knees were more posteriorly located compared with normal knees (MA TKA: from 10° to 20° of flexion; FA TKA: at 10° of flexion) (Figure 5). At higher flexion angles, both TKA knees were positioned more anteriorly compared with normal knees (MA TKA: beyond 70° of flexion; FA TKA: beyond 50° of flexion). In lateral LCPs, both TKA knees were more anteriorly located compared with normal knees (MA TKA: beyond 50° of flexion; FA TKA: beyond 30° of flexion) (Figure 5).

### ACL forces (Figure 6)

**Figure 6:**
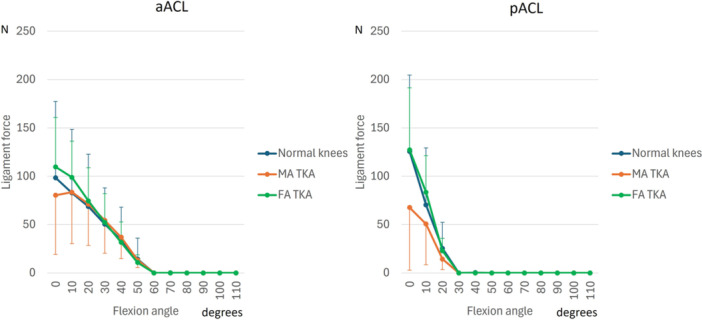
ACL force during squatting. ACL, anterior cruciate ligament.

Both aACL and pACL forces decreased with increasing knee flexion. No significant differences were observed between TKA knees and normal knees.

### PCL forces (Figure 7)

**Figure 7:**
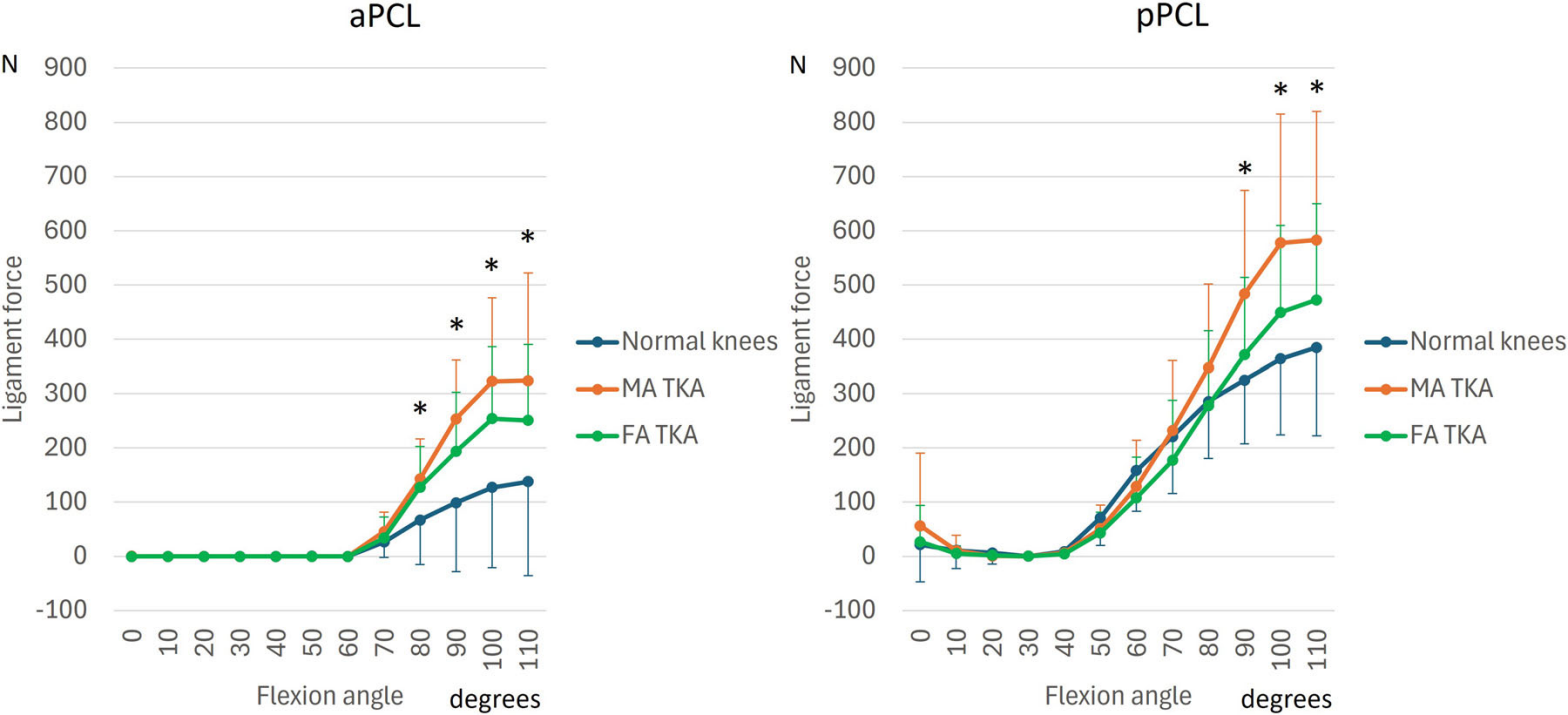
PCL force during squatting. *Significant differences between normal knees and MA TKA knees (*p* ≤ 0.05). APT, anteroposterior translation; MA TKA, mechanical alignment total knee arthroplasty; PCL, posterior cruciate ligament.

Both aPCL and pPCL tensions increased with knee flexion. Beyond 80° of flexion, aPCL tension in MA TKA knees was significantly greater than that in normal knees. Similarly, beyond 90° of flexion, pPCL tension in MA TKA knees exceeded that of normal knees.

## DISCUSSION

The key findings of this study indicate that PCL forces in MA TKA knees were significantly greater than those in normal knees. Tsai et al. reported that MA BCR‐TKA knees with a symmetrical articular surface exhibited significant PCL overstretching during flexion compared with contralateral native knees [39]. This observation suggests that in MA BCR‐TKA knees with a symmetrical articular surface, the PCL tightness might be more readily observed.

By contrast, ACL forces in BCR‐TKA knees were not significantly different from those in normal knees. An in vitro study reported that ACL in situ forces in MA BCR‐TKA knees were higher than those in intact knees during passive motion [26]. Additionally, a previous study demonstrated that ACL forces following MA BCR‐TKA were significantly greater than preoperative values [17]. These findings suggest that in vivo ACL forces during weight‐bearing high‐flexion activity were re‐tensioned by BCR‐TKA, approximating normal physiological conditions.

With regard to the APT of the LCPs, the posterior translation of both medial and lateral LCPs was reduced from mid‐flexion to high‐flexion compared with normal knees. Typically, medial and lateral menisci are removed during TKA. Previous studies have reported that menisci undergo substantial displacement and play a significant role in transmitting contact forces during deep flexion; in other words, menisci are essential stabilisers of AP translation [40, 43]. Furthermore, several studies have demonstrated that preoperative OA knees exhibit reduced posterior translation during knee flexion [8, 33]. These findings suggest that even with cruciate ligament preservation, BCR‐TKA does not fully restore native APT on the articular surface. Considering that FA TKA is positioned more anteriorly, it may more accurately reflect preoperative posterior tightness resulting from OA progression. A previous study reported that reduced lateral AP translation in MA BCR‐TKA was correlated with the PCL tension [17]. This observation suggests that diminished femoral rollback may contribute to the higher PCL force observed in MA TKA.

Unlike the APT of the LCPs, the APT of SEA in both BCR‐TKA closely resembled that of normal knees. SEA is generally considered to be closer to the flexion axis than the geometric centre axis [24]. By contrast, LCPs were more susceptible to the influence of the femoral articular surface. This prosthesis featured a multi‐radius femoral component designed to mimic healthy femoral condyles. However, the radius markedly decreased at 30°–40° of flexion, as indicated by the manufacturer [13]. Therefore, these findings suggest that the femoral articular surface may not fully replicate the geometry of the native femur.

In BCR‐TKA, greater external rotation was observed at full extension compared with normal knees. This trend was more pronounced in MA TKA than in FA TKA. A previous study demonstrated that patients with lower PROMs exhibited increased external rotation at extension following MA BCR‐TKA [22]. These findings suggest that the recreation of the screw‐home mechanism, facilitated by a functionally preserved ACL, is particularly important in MA TKA.

Both MA TKA and FA TKA knees exhibited less varus alignment compared with normal knees. Kage et al. reported that the FA TKA group demonstrated significantly greater varus alignment compared with the MA TKA group [15]. However, although FA TKA exhibited a slightly more physiological varus alignment, its effect on overall varus–valgus balance may be limited.

This study has some limitations. First, the follow‐up duration was relatively short, and kinematics and ligament forces at longer‐term follow‐up may differ from those reported here. Second, only patients capable of performing squatting activities were included, limiting the generalisability of our findings to individuals who are unable to perform such movements. Third, although ligament strain was assessed using CT, MRI, and fluoroscopy, ligament forces were estimated based on ligament strain values and previously published stiffness parameters [16, 35, 36]. Therefore, the calculated ligament forces may not reflect absolute in vivo forces for each patient. However, the relative comparisons presented in this study remain valid. Forth, the effect size in this study was not very small. Therefore, a type Ⅱ error may have influenced the detection of subtle differences.

## CONCLUSIONS

Cruciate ligament forces, particularly PCL forces, were significantly greater in MA TKA knees than in normal knees. However, FA TKA and MA TKA exhibited similar kinematics and changes in ACL force. Although FA TKA was able to more closely restore the physiological cruciate ligament balance, this effect appeared to be limited.

## AUTHOR CONTRIBUTIONS

*Conceptualisation*: Kenichi Kono and Hiroshi Inui. *Methodology*: Kenichi Kono, Hiroshi Inui, Takaharu Yamazaki, Darryl D'Lima. *Investigation*: Kenichi Kono and Tomofumi Kage. *Data curation*: Kenichi Kono, Hiroshi Inui, and Tomofumi Kage. *Writing—original draft*: Kenichi Kono. *Writing— review and editing*: Kenichi Kono. *Supervision*: Hiroshi Inui, Shuji Taketomi, Darryl D'Lima, Tetsuya Tomita, and Sakae Tanaka.

## CONFLICT OF INTEREST STATEMENT

The authors declare no conflicts of interest.

## ETHICS STATEMENT

This study was approved by the University of Tokyo Institutional Review Board under approval number 10462‐(2). Informed consent was obtained from all participants prior to the study enrolment.

## Data Availability

The datasets used and/or analysed during the current study are available from the corresponding author upon reasonable request.
